# Altered Functional Connectivity after Epileptic Seizure Revealed by Scalp EEG

**DOI:** 10.1155/2020/8851415

**Published:** 2020-11-24

**Authors:** Yi Liang, Chunli Chen, Fali Li, Dezhong Yao, Peng Xu, Liang Yu

**Affiliations:** ^1^Department of Neurology, Sichuan Academy of Medical Sciences and Sichuan Provincial People's Hospital, Chengdu 610072, China; ^2^Department of Neurology, Affiliated Hospital of University of Electronic Science and Technology of China, Chengdu 610072, China; ^3^The Clinical Hospital of Chengdu Brain Science Institute, MOE Key Lab for Neuroinformation, University of Electronic Science and Technology of China, Chengdu 611731, China; ^4^School of Life Science and Technology, Center for Information in Medicine, University of Electronic Science and Technology of China, Chengdu 611731, China

## Abstract

Epileptic seizures are considered to be a brain network dysfunction, and chronic recurrent seizures can cause severe brain damage. However, the functional brain network underlying recurrent epileptic seizures is still left unveiled. This study is aimed at exploring the differences in a related brain activity before and after chronic repetitive seizures by investigating the power spectral density (PSD), fuzzy entropy, and functional connectivity in epileptic patients. The PSD analysis revealed differences between the two states at local area, showing postseizure energy accumulation. Besides, the fuzzy entropies of preseizure in the frontal, central, and temporal regions are higher than that of postseizure. Additionally, attenuated long-range connectivity and enhanced local connectivity were also found. Moreover, significant correlations were found between network metrics (i.e., characteristic path length and clustering coefficient) and individual seizure number. The PSD, fuzzy entropy, and network analysis may indicate that the brain is gradually impaired along with the occurrence of epilepsy, and the accumulated effect of brain impairment is observed in individuals with consecutive epileptic bursts. The findings of this study may provide helpful insights into understanding the network mechanism underlying chronic recurrent epilepsy.

## 1. Introduction

Highly synchronized paroxysmal discharge in the brain causes epileptic seizures, along with loss of consciousness and movement dysfunction [1–3]. Previous studies focusing on epileptic seizures found increased synchronization [4–6] and increased energy of brain waves [7] occur before the onset of epileptic seizure. The power spectral density (PSD) has been widely used to measure the fluctuating power of related epileptic activity [8, 9]; for example, Bettus et al. estimated the PSD difference between drug-resistant and drug-sensitive mesial temporal lobe epilepsy patients and found significantly decreased subtheta PSD in the drug-resistant group [10]. Additionally, nonlinear analyses have been widely used to uncover the dynamic information of the brain activity in epileptic patients (EPs) [11]. For example, Li et al. proposed an automatic epilepsy detection approach, including entropy [12]. Entropy is a nonlinear index that reflects the degree of chaos within a system [13]. It is often used to analyze epileptic electroencephalogram (EEG) signals to detect whether there is an epileptic attack or to inspect the state of epileptic seizures [14]. At present, fuzzy entropy, an improvement of sample entropy, is widely used to measure the complexity of EEG signals [15] and to study epileptic seizures [16].

The brain works as a large-scale network [17], and efficient transmission and processing of related information depend on the efficient brain network [18, 19]. However, the deficits of certain regions disturb the processing of upcoming information in the brain, especially long-range couplings, consequently leading to network failure [20, 21]. In fact, epilepsy is described as a network disorder [22]. Network neuroscience [23] thus raises a ‘bridge' between epilepsy and brain network analysis [24–26]. Multiple neuroimaging techniques, including intracranial [27, 28] and scalp EEG [29, 30], as well as functional magnetic resonance imaging [31, 32], have been applied to investigate the mechanisms leading to epileptic seizures [6]. For example, Li et al. identified the transition of functional connectivity from inter- to preictal states and found that the preictal state caused fluctuations in related network properties, including decreased characteristic path length, increased clustering coefficient, and global and local efficiency [33].

Chronic recurrent seizures usually cause severe brain damage [34–36]. As illustrated, chronic recurring seizures cause continuing neural reorganization and progressive excitability of the brain. Epilepsy is self-perpetuating with each attack facilitating the occurrence of another by increasing the instability of neural elements [37]. Vlooswijk et al. found that patients with chronic epilepsy, compared to normal controls, experienced a disruption of both local segregation and global integration in the brain; and, they also found an association between pronounced intellectual decline and disturbed local segregation [38].

Although previous studies have investigated the cumulative brain damage after long-term epileptic seizures, few studies have further explored the mechanism of recurrent epileptic bursts (gradual process of an epileptic seizure), especially potential brain network mechanism. In this study, we thus hypothesized that seizures beget seizures, that is, every single epileptic seizure may cause slow alteration in the brain activity and such alteration may lead to the accumulation of seizures over time. To validate our hypothesis, corresponding functional networks, as well as the PSD and fuzzy entropy, of pre- and postseizure were constructed and then investigated in a group of ten EPs to uncover the mechanism underlying chronic recurrent epilepsy. Moreover, to validate our assumption, a correlation between network metrics and seizure number was further performed for one EP who experienced seizures during a 24 h hospital monitoring.

## 2. Materials and Methods

### 2.1. Participants

The protocols of this study was approved by the Medical Ethics Committee of Sichuan Academy of Medical Sciences & Sichuan Provincial People's Hospital, and written informed consent was obtained from all patients. Ten EPs were recruited in this study. Before 24 h EEG monitoring, all EPs were asked to suspend their antiepileptic drug treatment. Details of these patients were shown in Table 1.

### 2.2. EEG Recording

Sixteen-channel EEG (i.e., Fp1/2, F3/4/7/8, C3/4, P3/4, O1/2, and T3/4/5/6) were recorded by using the Australian COMPUMEDICS Greal series of digital video EEG with a sampling rate of 256 Hz. All of the sixteen electrodes were positioned according to 10-20 international electrode system. Electrocardiograms and electromyograms were also recorded by two extra electrodes. During the 24 h recording, single or dual cameras were also used to monitor the patients' behavior, and patients would experience at least one complete sleep-wake cycle, meanwhile patients had at least one episode of epileptic seizure. The starts and ends of each seizure episode were manually recorded by the physicians.

### 2.3. EEG Data Analysis

For each patient, 10 min before and 10 min after epileptic seizure EEG datasets were included in this study. For each 10 min EEG segment, we first rereferenced this segment to average reference. Then, to remove low-frequency drift and high-frequency noise, the data was bandpass filtered within a frequency range of (1, 45) Hz. And, we further segmented the filtered data into 6 s-length segment. Finally, a threshold of ±80 *μ*V was used to exclude segments still contaminated by high-amplitude artifacts.

#### 2.3.1. Linear and Nonlinear Analyses

PSD, which estimates the distribution of power spectrum over frequency bins, is a well-established technique for quantitative analysis of EEG signals, as well as for epilepsy [8, 39]. In this study, we first computed the PSD of pre- and postseizure via Welch's method and then statistically compared the PSD per electrode between the two conditions to uncover any potential differences using paired *t*-test.

Fuzzy entropy, a nonlinear measure, can describe the complexity of EEG signals [14], which was developed by Chen et al. in 2007 [40]. For short-time series contaminated by noise [41], fuzzy entropy is not sensitive to interference, but to change of information content [14]. Thus, fuzzy entropy has been widely used to study epileptic seizures [42, 43]. In the present study, we first calculated the fuzzy entropy of pre- and postseizure states and then statistically compared the fuzzy entropy per electrode between the two conditions to investigate any alteration in the brain activity using paired *t*-test.

#### 2.3.2. Functional Networks

Epileptic seizures are usually characterized by abnormal, high, synchronous neuronal discharges, which could effectively be measured in the frequency domain. In this study, we therefore employed coherence to measure the cooperative, synchrony-defined neuronal assemblies at each frequency bin. The coherence coefficient between signal *x* and *y* was calculated as follows:

(1) $$\begin{array}{c} C\left(f\right)=\frac{{\left|S_{xy}\left(f\right)\right|}^{2}}{S_{xx}\left(f\right)S_{yy}\left(f\right)}, \end{array}$$

where *C*(*f*) denotes the coherence coefficient of signal *x*(*t*) and *y*(*t*), *S*_*xy*_(*f*) is the cross-spectrum of signal *x*(*t*) and *y*(*t*); and *S*_*xx*_(*f*) and *S*_*yy*_(*f*) are the autospectrum of signals *x*(*t*) and *y*(*t*), respectively.

Any potential topological differences between the pre- and postseizure states were investigated by using paired *t*-test. Thereafter, based on the constructed coherence network, we calculated the corresponding network properties, including characteristic path length (CPL) and clustering coefficient (CC) in different frequency bands (i.e., delta, theta, alpha, beta, and low gamma). Theoretically, the CPL and CC are the parameters measuring the functional integration and separation of a given network, which indexes the ‘global' and ‘local' information processing of the network, respectively [44]. Both short CPL and high CC consistently reflect the efficiency resource allocation and index the high efficiency of information processing. Then, the any potential difference in information processing was uncovered by comparing these network metrics utilizing paired *t*-test. The two network properties were estimated as follows:

(2) $$\begin{array}{c} \text{CPL}=\frac{1}{n}\underset{i\in \psi }{\sum }\frac{\sum_{j\in \Psi ,j\neq i}d_{ij}}{n-1},\\ \text{CC}=\frac{1}{n}\underset{\text{i}\in \Psi }{\sum }\frac{\sum_{j,h\in \Psi }{\left(C_{ij}C_{ih}C_{jh}\right)}^{1/3}}{\sum_{j\in \Psi }C_{ij}\left(\sum_{j\in \Psi }C_{ij}-1\right)}. \end{array}$$

Herein, *d*_*ij*_ and *C*_*ij*_ represent the shortest weighted path length and coherence coefficient between the *i*th and *j*th network nodes, respectively; *n* represents the node number; and *ψ* represents the set of all network nodes.

#### 2.3.3. Individual-Level Network Analysis

During the 24 h monitoring, only one patient experienced four episodes of epileptic seizure. Therefore, we intended to explore the potential correlation between the network metrics and seizure number of this subject to probe if gradual impairment of brain functions were revealed in those consecutive epileptic bursts. In specific, for the four seizure bursts, eight segments were extracted, i.e., 10 min preseizure and 10 min postseizure pair for each burst, which were labeled as 1-pre, 1-post, 2-pre, 2-post, 3-pre, 3-post, 4-pre, and 4-post.

## 3. Results

### 3.1. Linear and Nonlinear Differences

**Figure 1:**
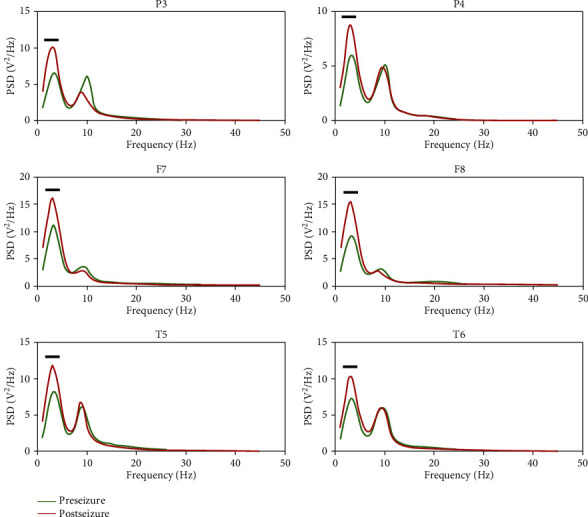
Grand-averaged PSD of the delta band pre- and postseizure. Green and red solid lines denote pre- and postseizure, respectively, and the thick black lines highlight significant PSD differences (*p* < 0.05) between the two states.

We first calculated and then compared the pre- and postseizure PSD of all channels at five bands, i.e., delta, theta, alpha, beta, and low gamma. As illustrated in Figure 1, six electrodes (i.e., P3, P4, F7, F8, T5, and T6) showed significant PSD differences (*p* < 0.05) in the delta band, indicating that the postseizure PSD was higher than the preseizure.

**Figure 2:**
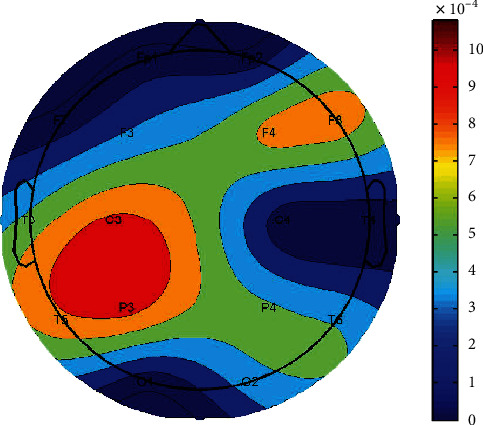
The topographic map of fuzzy entropy difference before and after seizure in the delta band. The red areas indicate that the differences are more significant.

Then, the corresponding fuzzy entropy of the two datasets was computed and compared. As displayed in Figure 2, in the delta band, the fuzzy entropies of preseizure in the frontal, central, and temporal regions are higher than that of postseizure (*p* < 0.01, fdr).

### 3.2. Functional Network

#### 3.2.1. Functional Connectivity

**Figure 3:**
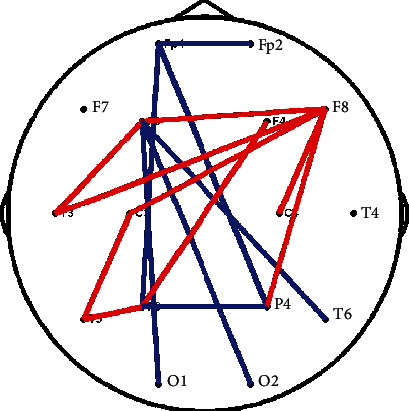
Topological differences between pre- and postseizure. The red and blue solid lines denote significantly (*p* < 0.05) enhanced and weakened functional connectivity postseizure, respectively.

Moreover, the topological differences in the delta band postseizure, as shown in Figure 3 (*p* < 0.05), exhibited decreased long-range connectivity (e.g., Fp1-P3/4 and F3-O1/2) but increased local connectivity (e.g., F3-T3, F8-C4, and C3-T5). However, no significant differences in network properties (i.e., CPL and CC) between the pre- and postseizure were found.

#### 3.2.2. Relationships between Network Metrics and Seizure Number

**Figure 4:**
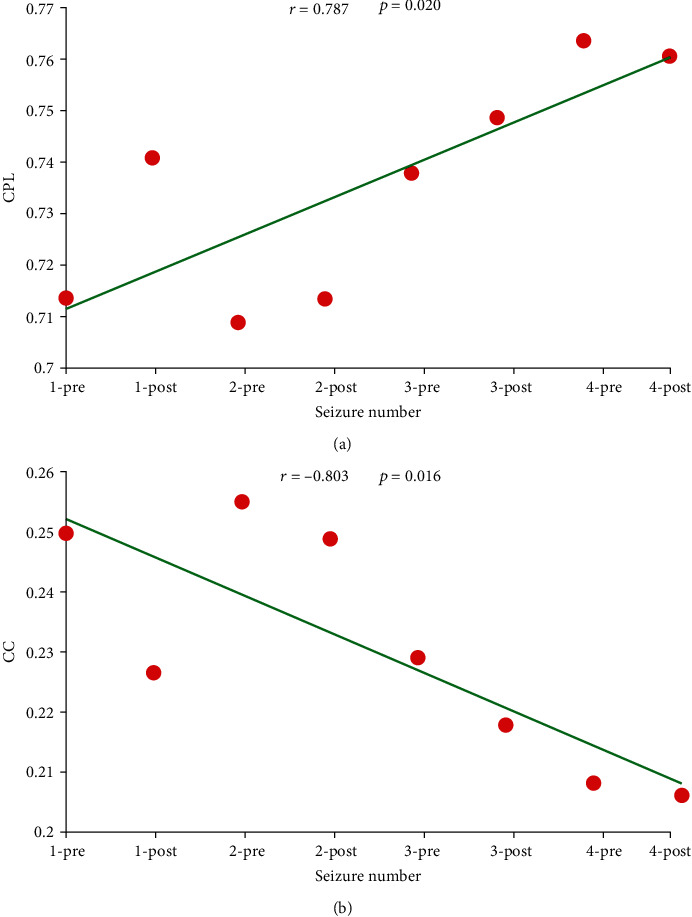
Potential relationships between network metrics and seizure number in the delta band. (a, b) The green solid line indicates the regression of network properties (CPL and CC) relative to the seizure number.

To further validate if the deficits of the brain network correlated with increasing number of epileptic bursts, the relationship between network metrics and seizure number of the patient who had four episodes of epileptic seizure was investigated. As shown in Figure 4, the characteristic path length in the delta band positively correlated with seizure number (*r* = 0.787, *p* = 0.020), while the clustering coefficient negatively correlated with seizure number (*r* = −0.803, *p* = 0.016).

## 4. Discussion

This study investigated the mechanism underlying chronic recurrent epilepsy by focusing on the brain networks before and after epileptic seizure. Our results show the mediation of regional epileptic networks in seizure episodes. Moreover, the correlation analysis of one patient with four episodes of recurrent seizures may indicate gradual impairment of brain functions with increment epileptic bursts.

As shown in Figure 1, six scalp electrodes (i.e., P3, P4, F7, F8, T5, and T6) showed increased postseizure PSD in the delta band, compared to the preseizure PSD, which may imply local energy accumulating after epilepsy burst. Epileptic activity can be regarded as a burst event of excessive energy resembling an earthquake or a volcanic eruption [45]. Due to such a characteristic of epileptic seizure as recurrence, there may be chronic abnormal activity or energy accumulation leading to the next seizure, which has been widely reported in previous studies [45, 46]. Osorio's study [47], which compared the seismic waves in 81,977 earthquakes and the brain waves in 16,032 epileptic seizures, showed that these two activities exhibited highly similar patterns and suggested that epileptic seizures can be metaphorically described as earthquakes of the brain. In the meantime, epileptic seizures are characterized by highly elevated frequencies and amplitudes of both electrical and magnetic signals [48, 49], and these high-frequency neural oscillations require elevated energy [50]. Of note, previous studies have revealed increased energy of delta waves before the onset of epileptic seizures [7]. For example, Rektor et al. found a significant increase in PSD during epileptic seizures, especially in slow band, such as delta waves; of note, even after seizures, there was a significant increase in PSD in all frequency ranges, including delta waves [51]. Moreover, there is evidence that the energy before and after epileptic seizures is mainly concentrated in the delta band [52]. Besides, the result of fuzzy epilepsy showed alteration in the brain activity before and after seizures. In detail, in the delta band, the fuzzy entropies of preseizure in the frontal, central, and temporal regions are higher than that of postseizure, which indicated that epileptic seizures cause attenuated brain flexibility, that is, the decrease in entropy indicates reduction in information processing at the cerebral cortex [53, 54]. In the meantime, we found a reduction in connections between epileptogenic zone and surrounding regions before epileptic seizure, as shown in Figure 3, which result in the isolation of epileptic neurons located in the epileptogenic zone [55]. The accumulative energy in epileptogenic zone after seizure may have exacerbated such isolation. This causes a reduction in the variability of the EEG signal, and therefore, results in lower entropy values [56]. In our present study, the increased synchronization among multiple electrodes had also been demonstrated before epileptic seizure; therefore, we assumed that, on the one hand, related energy of abnormal discharge first accumulated locally near the epileptogenic zone and then transmitted to other areas, which finally induced an epileptic seizure; on the other hand, the energy accumulation might result in the isolation of epileptic neurons near the epileptogenic zone, which leads to lower entropy values.

In this study, we assumed that the malfunction of local energy diffusion and its transmission to global areas not only resulted in the energy accumulation on local electrodes but also led to decreased functional network efficiency in the delta band. We further unveiled potential topological differences between pre- and postseizure states to validate network dysfunction. Actually, numerous studies have revealed abnormalities in brain networks. Our scalp EEG brain network exhibited attenuated long-range connectivity but enhanced local connectivity, as shown in Figure 3. Of note, results of our present study were consistent with previous studies that reported abnormalities in long-range connectivity also among the frontal, parietal, occipital lobes [57, 58]. For example, Luo et al. found decreased connectivity between the frontal and parietal lobes in absence epilepsy, when compared with healthy controls [59]. Meanwhile, the enhanced local connectivity coupled frontal and temporal lobes that had been diagnosed as the origin where abnormal discharge occurs. In fact, Bettus et al. have also reported the enhanced EEG connectivity in the epileptogenic zone [10]. To summarize, the decreased long-range connectivity among the frontal, parietal, and occipital lobes while enhanced local connectivity among the frontal and temporal lobes may reflect the impaired brain functions and abnormal synchronization among certain brain regions, which may result in another epileptic seizure.

Thereafter, to further validate our current results, the relationship between the network metrics and seizure number of the patient having four seizure bursts was investigated. And higher CPL and lower CC corresponding to the postseizure, compared to that of preseizure, were found (Figure 4). CPL is inversely related to the level of network integration, whereas CC is positively correlated with the level of network segregation, the decreased CC and prolonged CPL of postseizure might index the more severe brain dysconnectivity after epileptic seizure; additionally, both the positive relationship of CPL versus seizure number and the negative one between CC and seizure number might further conclude that the brain is gradually disrupted and impaired with consecutive epilepsy bursts, which could be also validated by the disrupted small-world organization in epileptic patients [25, 38, 60].

In summary, our present study demonstrated local energy accumulation and altered functional connectivity after epileptic seizure, as well. And gradual increment in brain deficits is also found to significantly correlate with patients' multiple consecutive epileptic bursts. These results help uncover potential mechanism accounting for the chronic recurrent epilepsy. However, since a relatively small patient size is included in our present study, to facilitate the clinical treatment and effective intervention of epilepsy, more patients will be recruited in the future. Meanwhile, another limitation of this study is that the impact of patient age is not taken into consideration. In our future work, when recruiting more epilepsy patients, their age range will be controlled to further validate our findings.

## Figures and Tables

**Table 1:** The detailed information about the EPs.

	EP1	EP2	EP3	EP4	EP5	EP6	EP7	EP8	EP9	EP10
Age (years)	61	33	56	37	19	36	48	22	30	10
Sex (M/F)	M	M	M	M	M	F	M	M	F	M
Seizure type	Focal	Focal	Focal	Focal	Focal	Focal	Focal	Focal	Focal	Focal
Duration (year)	1	16	7	10	3	1/6	1	20	26	1

## Data Availability

The data used to support the findings of this study are available from the corresponding author upon request.

## References

[1] Cheung M., Chan A. S., Chan Y., Lam J. M. K., Lam W. (2006). Effects of illness duration on memory processing of patients with temporal lobe epilepsy. *Epilepsia*.

[2] Blumenfeld H. (2012). Impaired consciousness in epilepsy. *Lancet Neurology*.

[3] Hommet C., Sauerwein H. C., De Toffol B., Lassonde M. (2006). Idiopathic epileptic syndromes and cognition. *Neuroscience & Biobehavioral Reviews*.

[4] van Quyen M. L., Navarro V., Baulac M., Renault B., Martinerie J. (2001). Anticipation of epileptic seizures from standard EEG recordings. *The Lancet*.

[5] Mormann F., Lehnertz K., David P., Elger C. E. (2000). Mean phase coherence as a measure for phase synchronization and its application to the EEG of epilepsy patients. *Physica D: Nonlinear Phenomena*.

[6] Zhang L., Liang Y., Li F. (2017). Time-varying networks of inter-ictal discharging reveal epileptogenic zone. *Frontiers in Computational Neuroscience*.

[7] Burns S. P., Santaniello S., Yaffe R. B. (2014). Network dynamics of the brain and influence of the epileptic seizure onset zone. *Proceedings of the National Academy of Sciences of the United States of America*.

[8] Al Ghayab H. R., Yan L., Siuly S., Shahab A. (2018). Epileptic EEG signal classification using optimum allocation based power spectral density estimation. *IET Signal Processing*.

[9] Pedersen M., Omidvarnia A., Curwood E. K., Walz J. M., Rayner G., Jackson G. D. (2017). The dynamics of functional connectivity in neocortical focal epilepsy. *NeuroImage: Clinical*.

[10] Bettus G., Wendling F., Guye M. (2008). Enhanced EEG functional connectivity in mesial temporal lobe epilepsy. *Epilepsy Research*.

[11] Lehnertz K. (2008). Epilepsy and nonlinear dynamics. *Journal of Biological Physics*.

[12] Li M., Chen W., Zhang T. (2016). Automatic epilepsy detection using wavelet-based nonlinear analysis and optimized SVM. *Biocybernetics & Biomedical Engineering*.

[13] Lemons D. S. (2013). *A Student's Guide to Entropy*.

[14] Acharya U. R., Fujita H., Sudarshan V. K., Bhat S., Koh J. E. W. (2015). Application of entropies for automated diagnosis of epilepsy using EEG signals: a review. *Knowledge-Based Systems*.

[15] Cao Z., Lin C. T. (2018). Inherent fuzzy entropy for the improvement of EEG complexity evaluation. *IEEE Transactions on Fuzzy Systems*.

[16] Xiang J., Li C., Li H. (2015). The detection of epileptic seizure signals based on fuzzy entropy. *Journal of Neuroscience Methods*.

[17] Park H., Friston K. J. (2013). Structural and functional brain networks: from connections to cognition. *Science*.

[18] Zhang R., Yao D., Valdés-Sosa P. A. (2015). Efficient resting-state EEG network facilitates motor imagery performance. *Journal of Neural Engineering*.

[19] Li F., Tao Q., Peng W. (2020). Inter-subject P300 variability relates to the efficiency of brain networks reconfigured from resting-to task-state: evidence from a simultaneous event-related EEG-fMRI study. *NeuroImage*.

[20] Engel J., Thompson P. M., Stern J. M., Staba R. J., Bragin A., Mody I. (2013). Connectomics and epilepsy. *Current Opinion in Neurology*.

[21] Englot D. J., Hinkley L. B., Kort N. S. (2015). Global and regional functional connectivity maps of neural oscillations in focal epilepsy. *Brain*.

[22] Kramer M. A., Cash S. S. (2012). Epilepsy as a disorder of cortical network organization. *The Neuroscientist*.

[23] Bassett D. S., Sporns O. (2017). Network neuroscience. *Nature Neuroscience*.

[24] Bartolomei F., Bettus G., Stam C. J., Guye M. (2013). Interictal network properties in mesial temporal lobe epilepsy: a graph theoretical study from intracerebral recordings. *Clinical Neurophysiology*.

[25] Liao W., Zhang Z., Pan Z. (2010). Altered functional connectivity and small-world in mesial temporal lobe epilepsy. *PLoS One*.

[26] Wang J., Qiu S., Xu Y. (2014). Graph theoretical analysis reveals disrupted topological properties of whole brain functional networks in temporal lobe epilepsy. *Clinical Neurophysiology*.

[27] Bandt S. K., Bundy D. T., Hawasli A. H. (2014). The role of resting state networks in focal neocortical seizures. *PLoS One*.

[28] Fuertinger S., Simonyan K., Sperling M. R., Sharan A., Hamzeisichani F. (2016). High-frequency brain networks undergo modular breakdown during epileptic seizures. *Epilepsia*.

[29] Storti S. F., Galazzo I. B., Khan S., Manganotti P., Menegaz G. (2017). Exploring the epileptic brain network using time-variant effective connectivity and graph theory. *IEEE Journal of Biomedical and Health Informatics*.

[30] Vecchio F., Miraglia F., Curcio G. (2015). Cortical connectivity in fronto-temporal focal epilepsy from EEG analysis: a study via graph theory. *Clinical Neurophysiology*.

[31] Liu F., Wang Y., Li M. (2017). Dynamic functional network connectivity in idiopathic generalized epilepsy with generalized tonic-clonic seizure. *Human Brain Mapping*.

[32] Xiao F., An D., Zhou D. (2017). Functional MRI-based connectivity analysis: a promising tool for the investigation of the pathophysiology and comorbidity of epilepsy. *Seizure*.

[33] Li F., Liang Y., Zhang L. (2019). Transition of brain networks from an interictal to a preictal state preceding a seizure revealed by scalp EEG network analysis. *Cognitive Neurodynamics*.

[34] Ben-Ari Y., Holmes G. L. (2006). Effects of seizures on developmental processes in the immature brain. *Lancet Neurology*.

[35] Liu Z., Yang Y., Silveira D. C. (1999). Consequences of recurrent seizures during early brain development. *Neuroscience*.

[36] Pitkanen A., Sutula T. P. (2002). Is epilepsy a progressive disorder? Prospects for new therapeutic approaches in temporal-lobe epilepsy. *Lancet Neurology*.

[37] Gowers W. R. (1901). *Epilepsy and Other Chronic Convulsive Diseases: Their Causes, Symptoms, and Treatment*.

[38] Vlooswijk M. C. G., Vaessen M. J., Jansen J. F. A. (2011). Loss of network efficiency associated with cognitive decline in chronic epilepsy. *Neurology*.

[39] Singh M., Kaur S. (2012). Feature selection for epilepsy detection using EEG. *International Journal of Information Technology and Knowledge Management*.

[40] Chen W., Wang Z., Xie H., Yu W. (2007). Characterization of surface EMG signal based on fuzzy entropy. *IEEE Transactions on Neural Systems and Rehabilitation Engineering*.

[41] Chen W., Zhuang J., Yu W., Wang Z. (2009). Measuring complexity using FuzzyEn, ApEn, and SampEn. *Medical Engineering & Physics*.

[42] Raghu S., Sriraam N., Kumar G. P., Hegde A. S. (2018). A novel approach for real-time recognition of epileptic seizures using minimum variance modified fuzzy entropy. *IEEE Transactions on Biomedical Engineering*.

[43] Tibdewal M. N., Dey H. R., Manjunatha M., Ray A., Malokar M. (2017). Multiple entropies performance measure for detection and localization of multi-channel epileptic EEG. *Biomedical Signal Processing and Control*.

[44] Rubinov M., Sporns O. (2010). Complex network measures of brain connectivity: uses and interpretations. *NeuroImage*.

[45] Wu Y., Liu D., Song Z. (2015). Neuronal networks and energy bursts in epilepsy. *Neuroscience*.

[46] Cloix J.-F., Hevor T. (2009). Epilepsy, regulation of brain energy metabolism and neurotransmission. *Current Medicinal Chemistry*.

[47] Osorio I., Frei M. G., Sornette D., Milton J., Lai Y.-C. (2010). Epileptic seizures: quakes of the brain?. *Physical Review E*.

[48] Nishida M., Juhasz C., Sood S., Chugani H. T., Asano E. (2008). Cortical glucose metabolism positively correlates with gamma-oscillations in nonlesional focal epilepsy. *NeuroImage*.

[49] Widjaja E., Shammas A., Vali R. (2013). FDG-PET and magnetoencephalography in presurgical workup of children with localization-related nonlesional epilepsy. *Epilepsia*.

[50] Lord L.-D., Expert P., Huckins J. F., Turkheimer F. E. (2013). Cerebral energy metabolism and the brain's functional network architecture: an integrative review. *Journal of Cerebral Blood Flow and Metabolism*.

[51] Rektor I., Kuba R., Brázdil M., Halámek J., Jurák P. (2011). Ictal and peri-ictal oscillations in the human basal ganglia in temporal lobe epilepsy. *Epilepsy & Behavior*.

[52] Omerhodzic I., Avdakovic S., Nuhanovic A., Dizdarevic K. (2013). Energy distribution of EEG signals: EEG signal wavelet-neural network classifier. http://arxiv.org/abs/1307.7897.

[53] Kannathal N., Choo M. L., Acharya U. R., Sadasivan P. K. (2005). Entropies for detection of epilepsy in EEG. *Computer Methods & Programs in Biomedicine*.

[54] Chua Chua K., Chandran V., Rajendra Acharya U., Lim C. M. (2011). Application of higher order spectra to identify epileptic EEG. *Journal of Medical Systems*.

[55] Petitmengin C., Baulac M., Navarro V. (2006). Seizure anticipation: are neurophenomenological approaches able to detect preictal symptoms?. *Epilepsy & Behavior*.

[56] Acharya U. R., Molinari F., Sree S. V., Chattopadhyay S., Ng K.-H., Suri J. S. (2012). Automated diagnosis of epileptic EEG using entropies. *Biomedical Signal Processing and Control*.

[57] Waites A. B., Briellmann R. S., Saling M. M., Abbott D. F., Jackson G. D. (2010). Functional connectivity networks are disrupted in left temporal lobe epilepsy. *Annals of Neurology*.

[58] Bettus G., Guedj E., Joyeux F. (2009). Decreased basal fMRI functional connectivity in epileptogenic networks and contralateral compensatory mechanisms. *Human Brain Mapping*.

[59] Luo C., Li Q., Lai Y. (2011). Altered functional connectivity in default mode network in absence epilepsy: a resting-state fMRI study. *Human Brain Mapping*.

[60] Bernhardt B. C., Chen Z., He Y., Evans A. C., Bernasconi N. (2011). Graph-theoretical analysis reveals disrupted small-world organization of cortical thickness correlation networks in temporal lobe epilepsy. *Cerebral Cortex*.

